# Application of the Activity-Based Costing Method for Unit-Cost Calculation in a Hospital

**DOI:** 10.5539/gjhs.v8n1p165

**Published:** 2015-05-15

**Authors:** Mahdi Javid, Mohammad Hadian, Hossein Ghaderi, Shahram Ghaffari, Masoud Salehi

**Affiliations:** 1 Department of Health Economics, School of Health Management and Information Science, Iran University of Medical Sciences, Tehran, Iran; 2 Department of Statistic and Mathematics, School of Health, Tehran University of Medical Sciences, Tehran, Iran

**Keywords:** activity-based costing, cost calculation, medical services

## Abstract

**Background:**

Choosing an appropriate accounting system for hospital has always been a challenge for hospital managers. Traditional cost system (TCS) causes cost distortions in hospital. Activity-based costing (ABC) method is a new and more effective cost system.

**Objective:**

This study aimed to compare ABC with TCS method in calculating the unit cost of medical services and to assess its applicability in Kashani Hospital, Shahrekord City, Iran.

**Methods:**

This cross-sectional study was performed on accounting data of Kashani Hospital in 2013. Data on accounting reports of 2012 and other relevant sources at the end of 2012 were included. To apply ABC method, the hospital was divided into several cost centers and five cost categories were defined: wage, equipment, space, material, and overhead costs. Then activity centers were defined. ABC method was performed into two phases. First, the total costs of cost centers were assigned to activities by using related cost factors. Then the costs of activities were divided to cost objects by using cost drivers. After determining the cost of objects, the cost price of medical services was calculated and compared with those obtained from TCS.

**Results:**

The Kashani Hospital had 81 physicians, 306 nurses, and 328 beds with the mean occupancy rate of 67.4% during 2012. Unit cost of medical services, cost price of occupancy bed per day, and cost per outpatient service were calculated. The total unit costs by ABC and TCS were respectively 187.95 and 137.70 USD, showing 50.34 USD more unit cost by ABC method. ABC method represented more accurate information on the major cost components.

**Conclusion:**

By utilizing ABC, hospital managers have a valuable accounting system that provides a true insight into the organizational costs of their department.

## 1. Introduction

The health sector is one of the most important parts of the service sector due to its impact on the protection as well as promotion of human life (Banerjee, Deaton, & Duflo, 2004). Hospitals face difficulties and challenges in balancing limited resources and costs to provide their demand for services (Gujral et al., 2010). One of the main challenges of hospital managers is development of cost information for decision-making and proper pricing of services. Hospital managers must provide healthcare services needed by the community at an acceptable level of quality and at the least possible cost; therefore, they need information on the actual cost of the services they provide.

Many hospitals, even those with new cost accounting systems, have difficulty in calculating actual direct and indirect costs for medical services (Meyer & Feingold, 1995), which necessitates attention to the acceptable costing systems.

There are two approaches in cost determination in accounting systems: traditional costing system (TCS) and activity-based costing system (ABC). The ABC method was developed to compensate for the deficiency of TCS, to allocate a cost driver suitable to any activity, and to calculate the cost price according to the activity. The TCS allocates some costs incurred in departments and uses a few cost drivers to allocate per capita costs. In contrast, ABC assigns resource costs to cost objects such as products, services, or customers based on the performed activities. It also offers correct outcomes compared with the TCS. Moreover, the ABC method is a two-stage process, because it assigns total costs first to activities and then to services (Ross, 2004).

Very few detailed studies have been performed on the economics of hospitals in middle-income and low-income countries (Adam & Evans, 2006; Mills, Kapalamula, & Chisimbi, 1993). Most of the studies have focused on individual units of hospital such as application of ABC in calculating cost price of surgery services (Baker & Boyd, 1997; Baratti et al., 2010; Chapko et al., 2009; Cinquini, Miolo Vitali, Pitzalis, & Campanale, 2009; Whiting, Martin, Zavala, & Hanto, 1999; Yereli, 2009), in hospital’s nursing station department (Popesko & Novák, 2011), and in radiology department (Canby, 1995). There are reports on successful applications of ABC to calculate cost price in medical services in many countries, eg, Turkey (Popesko & Novák, 2011), Japan (Cao, Toyabe, & Akazawa, 2006), Taiwan (Lin et al., 2007), India (Gujral et al., 2010), Spain (González, Quesada, Mack, & Urrutia, 2005), Brazil (de Carvalho Jericó & Castilho, 2010), the United States (Udpa, 1996), Sweden (Ridderstolpe, Johansson, Skau, Rutberg, & Ahlfeldt, 2002), and Iran (Rajabi & Dabiri, 2012).

In this study, was aimed to determine the cost price of medical services by ABC in Kashani Hospital, Shahrekord City, Iran, and compare the results with those of TCS.

## 2. Materials and Methods

This cross-sectional study was performed in Kashani hospital, Shahrekord City, Iran, in year 2013. All data were collected from the accounting reports, annual expenditure report, and medical record section of the Kashani Hospital, Shahrekord City, in 2012. The unit cost of medical services was calculated using the ABC method and all costs used in ABC program were expressed in the United States dollar (USD). Five cost categories were defined: wage, equipment, space, material, and overhead costs. Costs were divided into direct and indirect costs. The direct cost of each cost center was calculated by summing human resources, capital and materials costs. The indirect costs included all costs that could not be allocated directly to cost centers and were calculated using cost drivers. For staff who worked in more than one cost center, human resources costs were calculated based on the working hours in each cost center. Capital costs included annualized discounted depreciation of vehicles, building, equipment, and furniture. The depreciation cost was calculated using the straight-line depreciation approach. The steps taken to apply the ABC method were as follows:

**Step 1: Hospital Analysis and Cost Centers Classification**

The hospital was divided into several patients’ care cost centers (PCCs) and supportive cost centers (SCCs). The SCCs prepared facilities and offer services to all units such as management, kitchen, and accounting. The PCCs offered services to patients and included inpatients department, laboratory, and heart surgery. PCCs in inpatient units were defined according to the kind of provided service.

**Step 2: Identifying Major Activities in Hospital**

In this step, first, comprehensive distinction was made between two groups of activities: treatment-related activities, which were directly linked to inpatient units; and supporting activities, which supported the treatment process and the department.

We kept limited number of activities to avoid data overload. The treatment-related activities were identified by consulting team members. Supporting activities included department-supporting activities, ie, activities performed by personnel of inpatient department, and hospital-supporting activities.

**Step 3: Definition of Activity Cost Drivers**

In this step, we determined the cost driver for treatment-related and supporting activities. Cost driver rates of individual activities were calculated. Cost drivers were used to allocate indirect costs to cost centers and services. Some cost drivers were selected based on recommendations of experts of hospital management. The floor area was selected to distribute water, cleaning services, heating, and indirect electricity. The spent time per type of personnel for each activity and service was defined on the basis of interviews and time calculation.

**Step 4: Assigning Costs to Cost Center**

The first stage of the allocation process within the ABC system is assigning cost to cost centers. To allocate indirect costs, a resource cost driver must be determined. In this stage, costs were allocated to activity centers by using the following cost drivers: personnel workload, quantity of equipment, floor area, and estimation. Wage costs were allocated to the different activity groups on the basis of the percentage of time spent by a type of personnel for that activity.

**Step 5: Calculation the Unit Cost of Activity and Services**

The cost of administration center and consumed resources were assigned to products. The support costs were allocated to the products in this stage by using cost drivers. Moreover, the output of the given activity was determined.

In this step, the indirect costs were assigned to specific activities, and the activity costs were assigned to cost object. For each activity, the required materials as well as labor time were determined according to the structured interviews with involved person. The costs of each part of the activity were summed to form the cost of the activity:

Activity cost = cost of floor area + cost of materials + staff costs + cost of capital equipment + cost of hospital infrastructure + other indirect costs.

Final step in ABC application was the calculation of the cost price of every bed occupied in each day and the cost of other selected medical services.

The main outcome measure of study was determining cost units in different part of the hospital through both ABC and TCS methods and comparing the crude final values in USD. To calculating the cost price of bed occupancy day, we also calculated the unit cost of some other services.

The data were obtained from the final costs of the hospital at the end of year 2012. ABC and TCS methods were used to determine the costs from the available crude data. SPSS 14 (SPSS Inc, Chicago, Illinois, the United States) was used to assess differences between two methods through student’s t test. A P value < 0.05 was considered statistically significant.

## 3. Results

The staffing and output of Kashani Hospital are presented in Table 1. Regarding the application of ABC steps, the two-side allocation method was used for cost allocation. Table 2 shows cost price of some specific medical services.

**Table 1:** Staffing and Bed Occupancy Rate in Kashani Hospital^a^

Cost Centers	Doctors, NO.	Nurses, NO.	Beds, No.	Occupancy Rate, %
**Burn unit**	2	12	13	66.66
**CCU**	5	18	9	57.47
**Emergency ICU**	3	12	4	68.1
**Emergency**	12	77	30	100
**ENT**	4	12	20	46.9
**Heart Surgery & Neurosurgery**	6	19	28	68.34
**ICU**	8	23	9	64.57
**IPD eye**	3	12	18	31.74
**Men’s internal medicine**	4	15	28	59.5
**Men’s orthopedics**	6	17	27	70.24
**Men’s surgical unit**	6	14	24	58.33
**Neurology**	5	20	23	84.21
**Women’s internal medicine**	3	15	25	58.8
**Women’s orthopedics**	6	14	25	38.46
**Women’s surgical unit**	5	16	25	60.1
**Urology**	3	10	20	53
**Total**	81	306	328	67.4

a Abbreviations: ICU, intensive care unit; CCU, cardiac care unit; IPD, inpatient department; and ENT, ear, nose, throat.

**Table 2:** Unit Cost and Contribution of Different Cost Components in Total Costs of Selected Cost Centers

Cost Center	Unit	Human Resource, %	Capital Cost, %	Material Cost, %	Unit Cost, USD
**Biochemical Laboratory**	Examination	34.3	19.6	46.1	0.65
**Microbiology Laboratory**	Examination	37.1	12.1	50.8	3.25
**Ophthalmology Clinic**	Visit	63.9	16.05	20.05	5.85
**Orthopedic Clinic**	Visit	59	17.6	23.4	4.03
**Pharmacy**	Prescription	61.2	18.4	20.4	1.35
**Radiology**					
**Bone & soft tissue**	Examination	47.5	29.1	23.4	5.27
**CNS^a^**	Examination	47.5	29.1	23.4	4.02
**Head and neck**	Examination	47.5	29.1	23.4	6.02
**Lung**	Examination	47.5	29.1	23.4	4.43
**Urology**	Examination	47.5	29.1	23.4	3.81

a Abbreviation: CNS, central nervous system.

The cost price of occupancy bed-day for selected units is shown in Table 3. The cost for bed occupancy day ranged from 28.85 USD to 81.48 USD. The cost price of bed occupancy per day was 29.21 USD in the emergency department, 76.57 USD in cardiac care unit (CCU), and 33.61 USD in Men’s surgery unit. The distribution of the cost inputs of the Women’s surgery department were wage costs (50.68%), equipment costs (21.33%), per capita costs (19.15%), material costs (2.83%), and space costs (6.01%). Drugs and medical supplies accounted for 30% of the total materials cost of the inpatient department (IPD).

**Table 3:** Cost Price of Occupancy Bed-Day by Activity-Based Costing Method^a^

Cost Center	Cost Price of Occupancy Bed-Day, USD		

	Direct Cost	Indirect Cost	Total Cost
**Burn Unit**	19.39	18.49	37.88
**CCU**	61.44	22.04	81.48
**Ear, Throat, Nose**	29.14	31.66	60.8
**Emergency**	11.07	18.14	29.21
**Emergency ICU**	39.89	12.01	51.9
**Heart & Neurosurgery**	44.72	19.09	63.81
**ICU**	54.5	22.07	76.57
**Men’s Internal Medicine**	31.62	20.21	51.83
**Men’s Orthopedics**	29.33	20.19	49.52
**Men’s Surgery Unit**	20.16	13.45	33.61
**Women’s Internal Medicine**	25.17	18.77	43.94
**Women’s Orthopedics**	23.03	18.7	41.73
**Women’s Surgery Unit**	16.08	12.77	28.85
**Ophthalmology IPD**	17.03	22.33	39.35

a Abbreviations: CCU, cardiac care unit; ICU, intensive care unit; IPD, inpatient department; and ENT, ear, nose, throat.

**Figure 1:**
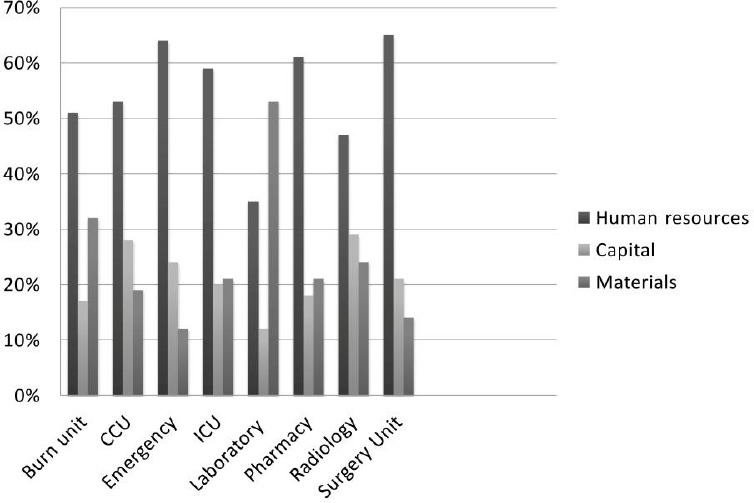
Contribution of Different Cost Components in Total Costs of Selected Cost Centers

The calculation showed that human resources were the largest component of hospitals’ total cost. More than 59% of the total costs of Kashani Hospital belonged to personnel costs of employee, which included the labor costs of doctors and nurses, the labor costs of the laboratory and other departments, operational and administrative staff. Tools, equipment, and material accounted for approximately 9% of the total costs. Around 8% of the total costs were drug costs (Figure 1). The results of ABC and TCS calculation are represented in Table 4. Implemented and the calculation were reported in the first part the ABC method, the result of TCS was reported in the second part.

**Table 4:** Unit Cost of Selected Medical Service by Activity-Based Costing and Traditional Cost System Methods^a,b^

Cost Center	Unit Cost By ABC, USD	Unit Cost With TCS, USD	Mean Differences, USD	P Value
**CCU**	81.48	66.66	-14.82	0.54
**Emergency**	29.21	19.20	-10.10	0.77
**Laboratory ^c^**	2.05	1.01	-1.04	0.004
**Ophthalmology IPD**	39.35	29	-10.35	< 0.001
**Pharmacy**	1.35	0.95	-0.40	0.002
**Radiology ^c^**	4.01	1.79	-2.22	0.59
**Surgery Unit**	30.50	19.09	-11.41	< 0.001
**Total**	187.95	137.70	-50.34	0.04

a Abbreviations: ABC, activity-based costing; TCS, traditional cost system; CCU, cardiac care unit; and IPD, inpatient department.

b The presented values are crude number derived from accountant data of Kashani Hospital at the end of 2012.

c Including all laboratories and radiology sections.

Comparing the result of ABC and TCS revealed that a significant difference between the TCS and ABC results; ABC system assigned lower costs for occupancy bed-day. On the other hand, cost price of occupancy bed-day should be less than the TCS by 30%.

## 4. Discussion

The results obtained from ABC were significantly different from those obtained from TCS in Kashani Hospital. In Kashani Hospital, unit cost of medical services is conventionally determined by TCS, which is not efficient because the costs do not reflect real costs. In TCS system, costs are directly allocated to services and overhead costs are allocated to cost object based on the patient-days. ABC system constitutes the cause-and-effect relationship between activities and cost objects and provides information on the actual unit cost of providing clinical services in hospital.

According to our results, application of ABC system in Kashani Hospital entails a number of benefits including the ability to quantify the actual costs of activities and identifying the relationship between the costs and activities. Several studies have shown that the ABC system was an applicable system for costing in hospitals (Duffy & McCahey, 2008; Ghaffari, Mohamadzadeh, Akbari, Salem Safi, & Yousefi, 2013; Udpa, 1996). One of the studies was implemented in a hospital in London in which the authors required to test ABC method with time driver in the hospital environment. The management of that hospital was under pressure, because some detachment of clinic was forfeit and ABC has shown to be a suitable cost system for that hospital (Demeere, Stouthuysen, & Roodhooft, 2009).

By using the ABC, we found that human resources were the largest component of total cost in Kashani Hospital (Figure 1). Several international studies on hospital costing also found that human resources constituted the majority of hospitals’ total cost (Kruk et al., 2010; Minh et al., 2010; Olukoga, 2007). We also found that 29% of total cost of Kashani Hospital was spent on the outpatient departments. Inpatient departments consumed more resources (48%) than outpatient services did. In a study by Grandlich et al. (Grandlich, 2004), total cost of inpatient departments significantly differed from that of outpatient departments in hospitals. One of the studies, examining organizing wide application of ABC in hospital, was published by Upda (Udpa, 1996) who examined the hospital inpatient services and stated that outpatient unit involves the much larger number of units of service with relatively small cost per unit. In radiology and laboratory sections, the cost of equipment and material is a significant portion of the total capital costs. Drug and medical supplies consume 41% of total material cost in the hospital. Therefore, the hospital managers might seek efficient ways of purchasing drug and medical supplies. The ABC model in our study was setup for different inpatient departments, based on the cost (Table 3). The cost price of occupancy bed-day in CCU significantly differed from that of other inpatient departments. The high costs might be due to low rate of occupied bed-day (57.47%) and high costs of wages.

## 5. Conclusion

Accurate unit cost of medical service is critical to improve efficiency and transparency in the hospital. According to the results of our study, Kashani Hospital managers should pay special attention to the results of ABC method. Unit cost calculation and ABC information clearly improved understanding of hospital managers about the different organizational processes and organizational unused capacity resources.
